# Attenuation of YAP1 can potentially target cancer stem cells to overcome drug resistance

**DOI:** 10.18632/oncoscience.449

**Published:** 2018-08-22

**Authors:** Kazuto Harada, Shumei Song, Jaffer A. Ajani

**Affiliations:** Jaffer A. Ajani: Department of Gastrointestinal Medical Oncology, The University of Texas MD Anderson Cancer Center, Houston, TX 77030, USA

**Keywords:** YAP1, hippo pathway, cancer stem cells, drug resistance, personalized therapy

The Hippo pathway was initially identified in *Drosophila* study; deletion of Hippo pathway caused dramatic over growth of various organs [1]. Then, the Hippo pathway, identified as tumor suppressors in human, have a key role in regulating organ development, tumorigenesis, tissue regeneration, and stem cell self- renewal [2]. One of the key factors of the Hippo pathway is Yes-associated protein 1 (YAP1), transcriptional coactivator interacting with TEA domain family members (TEAD). When the Hippo pathway is activated, YAP1 is phosphorylated and degraded in the cytoplasm. Once the Hippo pathway is turned off, YAP-1 translocates into the nucleus from cytoplasm and binds to TEAD (the transcription factor), leading to an increase transcription of the target genes [2]. Several factors have been discovered to regulate the Hippo pathway [2]. For instance, activation of G protein-coupled receptors activates YAP1 through inhibiting large tumor suppressor 1/2 (LATS1/2), which are kinases to phosphorylate YAP1. Moreover, the Wnt pathway has been discovered to crosstalk with YAP1. Cell-cell contact or extracellular matrix (ECM) also are involved in YAP1 activity. The Hippo pathway is activated in high cell density. On the other hand, some mediators from ECM, such as the integrin pathway, suppresses the Hippo pathway. Oxidative stress and cell metabolism are also interact with the Hippo pathway.

YAP1 overexpression and its contribution to cancer initiation and growth were reported in several cancer types [3]. YAP1 activation induces aggressive malignant behavior, such as epithelial-to-mesenchymal transition (EMT), proliferation, drug resistance, tumor-promoting microenvironment, and metastases. The YAP1-TEAD components facilitate target gene transcription related to the mesenchymal markers, such as vimentin and N-cadherin. Moreover, YAP1 activation is essential for EMT induced by the TGF-β-SMAD pathway. Song and colleagues reported that YAP1 promotes epidermal growth factor receptor (EGFR) expression, conferring chemoresistance in esophageal adenocarcinoma (EAC) cell line [4]. Several researches also reported that overexpression of YAP1 was associated with poor patient prognosis and advanced stage in lung, breast, liver, esophageal, and stomach cancer [3].

Importantly, several studies have demonstrated that YAP1 is correlated with cancer stem cells (CSCs) like properties [2]. CSCs are considered to contribute EMT, high metastatic potential, and therapy resistance [5]. CSCs were defined as a subset of cell population within a tumor that possesses the capacity to self-renew and to give rise to the heterogeneous lineages of cancer cells that comprise the tumor [6]. For instance, Song and colleagues found that YAP1 was overexpressed in EAC and regulated the transcription of SOX9, contributing to increasing CSCs properties [7]. Direct evidence from our laboratory suggests that YAP1 endows self-renewal capacity to non tumorgenic cells and tumor cells in EAC. Moreover, currently available treatment modalities target most mature and proliferating tumor cells and may lead to enrichment of the highly tumorigenic and drug resistant CSCs. Therefore, attenuation of YAP1 might be very important to reduce the density of CSCs and to overcome therapy resistance.

There are multiple upstream signals and pathways that may serve as novel therapeutic targets to inhibit YAP1. Verteporfin or VGLL4, that is commonly used as YAP inhibitor in research laboratories, binds to the YAP-TEAD complex and inhibits its transcription ability. Recently, Song and colleagues evaluated the new YAP1 inhibitor, CA3, and demonstrated that CA3 strongly inhibited YAP- TEAD activity and YAP-1 expression compared with other YAP1 inhibitors in EAC [8]. Especially, this phenomenon was more effective against radiation-resistant EAC cells that possess CSCs property. This result suggests that inhibiting YAP1 could be an effective therapy against CSCs, and thus have potential to overcome therapy resistance. However, agents against the Hippo pathway are under development.

We acknowledge that the Hippo pathway or function of YAP1 have not been completely understood at present and more research is warranted. First, the mechanism by which YAP1 controls expression level of the target genes remains unclear. Many factors and pathways are involved in the control of YAP1 activity, and thus unknown activator are yet to be uncovered. It is also unclear that when the Hippo pathway is turned off and YAP is activated, during carcinogenesis. Secondly, YAP1 activation in normal tissue has the key role in cell trans- differentiation, but in cancer tissue, there may be different signals for YAP1 activation to induce CSCs features.

In summary, the Hippo pathway has a key role in CSC capabilities, EMT, therapy resistance, and metastatic potential. Identification of unique markers of CSCs is important for diagnosis, treatment, and monitoring various cancers. YAP1 is a potential biomarker of CSC and a therapeutic target. Yap1 should be a major focus of cancer research.
